# Unmet Needs in the Treatment of RA in the Era of Jak-i: IDRA (Italian Delphi Rheumatoid Arthritis) Consensus

**DOI:** 10.1155/2018/3878953

**Published:** 2018-09-09

**Authors:** R. Caporali, A. Doria, G. F. Ferraccioli, P. L. Meroni, D. Zavaglia, F. Iannone

**Affiliations:** ^1^Division of Rheumatology, University of Pavia and IRCCS Policlinico S. Matteo Foundation, Pavia, Italy; ^2^Division of Rheumatology, University of Padova, Italy; ^3^Institute of Rheumatology and Affine Sciences, School of Medicine, Catholic University of the Sacred Heart, Rome, Italy; ^4^Immunorheumatology Research Laboratory, Istituto Auxologico Italiano, Milan, Italy; ^5^Medical Department, Pfizer Italia, Rome, Italy; ^6^DETO-Rheumatology Unit, University of Bari, Italy

## Abstract

Rheumatoid arthritis is the most common autoimmune arthritis in adult population. This disease is characterized by joint damage and systemic involvement that lead to general physical and mental impairment with consequent worsening of quality of life. Rheumatoid arthritis is also associated with a large economic burden to healthcare systems. The evidence from the literature indicates that, despite available treatments, several unmet needs still interfere with rheumatoid arthritis management. Based on this evidence, some of the unmet medical needs currently present in the management of the rheumatoid arthritis were identified and a Delphi questionnaire was submitted to 60 Italian Rheumatologists. The aim of this Delphi was to achieve a broad consensus on the most relevant* unmet needs *identified, in order to present the Italian reality in view of the availability of new molecules that could provide an effective therapeutic option in the treatment of patients with rheumatoid arthritis.

## 1. Introduction

Rheumatoid arthritis (RA) is the most common autoimmune arthritis in adult population, with an estimated prevalence of 0.3-1% worldwide. [1] An analysis of Italian epidemiological data shows an overall prevalence of 0.48%. [2]

Joint damage and systemic involvement of RA lead to general impairment of physical and mental, as well as of psychological wellbeing, with a consequent worsening of quality of life (QoL). [1] Furthermore, RA is associated with a large economic burden to the healthcare systems. In particular, persistence of pain, loss of productivity, and impairment of quality of life are linked to an increase of indirect and intangible costs of the disease. [1]

The evidence from the literature indicates that, despite available treatments, several unmet needs still interfere with RA management [3, 4]. Concerning symptoms, many patients with RA experience unbearable levels of pain and are not satisfied with their physical functioning outcomes, despite ongoing treatment [1]. In the longitudinal observational BARFOT study (Better Anti-Rheumatic PharmacOTherapy), patients with RA diagnosed in the 1990s were compared with those diagnosed in the 2000s; no differences between groups were found with regard to pain, patients' global health, and functional disability (assessed with HAQ) despite the new available therapies [5]. Furthermore, many patients with RA still experience unacceptable levels of fatigue even during intensive treatment. Clinical trials have demonstrated that even biologics in combination with MTX do not lead to significant improvements in fatigue. Fatigue is a determinant of quality of life and it continues to have a negative impact on more than 50% of patients with RA [1]. Together with physical functioning, mental health is often impaired in patients with RA. Both mental and physical impairment have a negative impact on working ability. Thirty percent of patients leave work prematurely due to the burden of the illness and, after 5 years from diagnosis, 30-40% of RA patients manifest disability. [1]

Remission is the primary target in RA management, even though there is a high heterogeneity in the definition of disease remission in clinical practice. In addition to the concepts of clinical and radiological remission, the new insight into tissue and molecular heterogeneity and the presence of nonresponders to available therapies emphasizes the importance of also including the concept of persistently active disease [6] that needs to be overcome by the new treatments.

The EULAR guidelines recommend MTX as first-line treatment in monotherapy and if response is not achieved, a switch to biologics or combination of conventional therapies is recommended. [7] Combination therapy with TNF inhibitors (TNFi) and csDMARD (conventional synthetic DMARDs) is more effective than TNFi in monotherapy both in MTX naïve patients and in those who fail MTX therapy, while maintaining the same safety profile. Importantly combination therapy reduces immunogenicity and progression of structural damage. However, this varies considerably among the different biologics. [8] In clinical practice, approximately a third of RA patients do not use combination therapy and take bDMARD only, as a monotherapy; the reasons are mainly due to intolerance and low adherence to MTX or other csDMARD [8].

Low adherence may lead to suboptimal response to treatment, late recovery, disease progression, and the need for a more aggressive therapy with the risk of adverse events. [9]. Several elements could have an impact on adherence. Therapeutic strategies based on more than three DMARDs are associated with a higher risk of low adherence and low persistence on therapy, compared to monotherapy with MTX (P≤ 0.02) [8].

Regardless of adherence, clinical response to treatment varies between individuals and it is not easy to predict. The underlying mechanisms of nonresponse are probably linked to the heterogeneity of the disease itself. Heterogeneity is not only clinical but also linked to the pathogenic pathways responsible for the disease in a given patient. For this reason, the need to develop predictive instruments of therapeutic response and personalized therapies, choosing the most efficacious and safest drug, for each patient even in long-term treatment, has been highlighted [10].

Based on this evidence, we identified some of the unmet medical needs currently present in the management of the RA. The aim of this project was to achieve a consensus within a group of expert Italian rheumatologists on the most relevant* unmet needs *identified, in order to depict the Italian reality in view of the availability of new molecules that could provide an effective therapeutic option in the treatment of patients with RA.

## 2. Methods

A stepwise approach was used:

- A selection of the relevant literature of the last 10 years, concerning unmet medical needs in the management of the RA, was carried out. This selection was shared among and validated by a board of experts. The board included clinicians with several years' experience in the field of RA from different Italian areas and, hence, representative of the Italian rheumatology community. Among the selected papers, the most relevant* unmet needs,* recognized by the international scientific community, were identified in the following 9 areas: (1) optimal pain control; (2) significant fatigue improvement; (3) satisfactory levels of physical functioning; (4) maintain workability; (5) social functioning; (6) treatment adherence; (7) prognostic factors of treatment response; (8) best conditions for an effective monotherapy; (9) disease remission.
- A first Delphi (1) round among the board components: the board ranked the identified areas according to a 5-point Likert scale, based on research evidence and their clinical judgment. In each area, a list of statements was identified. Finally, the list of the proposed statements was reviewed and approved, to then submit to six rheumatologists, experts in RA treatment. After the evaluators' feedback and comments, the final questionnaire was prepared, where each unmet need was detailed in 1 or 2 statements and each statement was then extended into 4 or more specific items, as specified in the results section. (Tables 1–5)
- A second Delphi round: The questionnaire was then sent to 60 Italian rheumatologists, with consolidated experience in RA treatment, using a modified Delphi method [1], in order to reach a consensus. All the experts were requested to have been working in a tertiary rheumatology center, having full access to all the marketed biologic drugs, being authors or coauthor of papers dealing with RA published on peer-reviewed journals over the last 15 years. They expressed their level of agreement according to the following 5-point Likert scale: 1 = strongly disagree, 2 = disagree, 3 = agree, 4 = more than agree, and 5 = strongly agree. Consensus was reached when the sum of items 1 and 2 (Disagree) or that of 3, 4, and 5 (Agree) reached 66%. No consensus was reached, when the sum of the responses for a negative consensus (1 and 2) or a positive consensus (3, 4, and 5) was <66%.

## 3. Results

Forty-eight out of the 60 questionnaires sent were completed. Panelists' agreement on statements was very high from the very beginning of the Delphi Study.

Answers to the Delphi questionnaire are shown in Tables 1–5.

Consensus was reached for the majority of the proposed statements. However, neither positive nor negative consensus was found in three cases (items 4.5, 8.3, and 8.4). (Tables 2 and 4) This may reflect a shared common view of Italian rheumatologists on the topics. In few cases consensus was reached with a lower percentage of agreement.

The experts involved in the study agreed that, for optimal pain control, a drug therapy, based on DMARDs and anti-inflammatories (associated with corticosteroids) and psychological/educational support, is both necessary.

Participants agreed that, in order to optimize physical functioning, the treat to target therapy is needed. However, they did not agree with regard to the chronic use of low doses of corticosteroids in association with DMARDs. This reflects the EULAR recommendations.

As far as the prognostic factors are concerned, the interviewed clinicians mainly focused on clinical factors. In fact, the consensus was not reached on the use of specific biomarkers (SNPs, micro-RNA, polymorphisms, etc.) or on the possible presence of antidrug antibodies if the patient was a secondary failure.

With regard to monotherapy, a lesser degree of agreement was reached on the use of bDMARD monotherapy or tsDMARD (targeted synthetic DMARDs) to remedy the high proportion of patients who withdraw from MTX intake.

Concerning the concept of remission, participants identified all criteria proposed (clinical, radiological, and immunological) as criteria to be reached in order to achieve complete remission.

Regarding the remission criteria used in clinical practice, almost all clinicians agree on the use of SDAI or CDAI and DAS. They also agree on the use of composite criteria but to a lesser extent.

## 4. Discussion

The primary goal of this Delphi consensus study was to identify the unmet medical needs in the management of patients with RA in Italy. The analysis of literature pertaining to this topic highlighted that, despite recent advances in both diagnosis and treatment of rheumatic diseases, several unmet needs are still present in RA management [1, 3, 4].

The results of this Delphi consensus confirm that the above evidence is also applicable to Italy. Consensus was reached for almost all statements with few exceptions. Statements that did not reach consensus (4.5, 8.3, and 8.4) (Tables 2 and 4) will be discussed below. Furthermore, some controversial aspects in the management of RA that have reached a lower degree of consensus will be discussed (10.3) (Table 5).

*Physical Functioning (Table 2). *Statement 4.6. Physical functioning levels depend on disease activity, structural damage, and fatigue. Current therapies do not often provide satisfactory levels of physical functioning. Therefore, to improve physical function, I always consider adding glucocorticoid at a low dose to DMARDs treatment.

The answers to the questionnaire were consistent with EULAR guidelines that recommend that short-term glucocorticoids should be considered when initiating or changing csDMARDs but should be tapered as rapidly as clinically feasible, due to the potential risk of adverse events [7].

The use of steroids in combination with csDMARD is justified as they have a supportive role for the efficacy of csDMARD in the early stages of treatment, until the csDMARD reaches its maximum effect. With the availability of molecules characterized by faster action, it can be hypothesized that there will be changes in the use of steroids in patients with RA. The most recent EULAR guidelines already state that glucocorticoids are typically not needed as a bridging therapy when drugs with a rapid onset of action (bDMARDs or tsDMARDs) are used and their association can increase the risk of infections [7]. Only clinical practice, in the coming years, will confirm this hypothesis.

*Prognostic Factors for Treatment Response (Table 4). *Statements 8.3 and 8.4. There are no clear prognostic factors for response to therapies. In the choice of treatment, I rely on the prevalent biological pathway to choose the most suitable MoA drug by searching for specific biomarkers (SNPs, micro-RNA, polymorphisms, etc.) and the possible presence of anti-drug antibodies if the patient is a secondary failure

Regarding the identification of prognostic factors and mechanisms underlying therapeutic failure, some studies, especially on TNF-is, have suggested that individual patient's characteristics and disease features could predict response to treatment [11, 12]. However, the conclusions of available studies are not univocal; in some cases, a correlation between clinical features and response to treatment has not been found [13]. Therefore, other factors could have an impact on clinical response particularly with biological treatment, among these immunogenicity. However, this has not been corroborated by the evidence available today [14–17]. Italian rheumatologists strongly agree with the choice of patients' characteristics (age, sex, concomitant therapies, body mass index, life habits, and comorbidity) as prognostic factors for treatment response. However, with regards to the use of biological biomarkers [SNPs, micro-RNA, polymorphisms, and evaluation of antidrug antibodies (ADA)] consensus has not been reached, even though 60% of the participants agreed with this item. In fact, Italian rheumatologists have a growing interest for these complex areas, despite the difficulty in their use and the absence of validation of these parameters. Consistent with the Delphi results, the 2016 Target Therapy Meeting included the identification of biomarkers within target tissues as one of the primary unmet needs in RA [4].

Concerning ADA, a recent review of the literature highlights that evidence-based recommendations on how to guide biologic therapy on the basis of drug levels and immunogenicity are lacking and that none of the assays available to detect ADAs are acceptable for routine clinical use. Therefore, according to the authors, the use of ADA seems to be indicated essentially to modulate dosage and route of administration of the drug [18]. In the light of these considerations and taking into account the availability of the large number of drugs with different mechanisms of action, changing the therapeutic strategy can be an acceptable choice in a real clinical setting of secondary failures.

*Disease Remission (Table 5). *Statement 10.3. The “treat to target” approach has been proposed since 2010 for RA patients, with remission as the best treatment outcome possible. However, high heterogeneity in the definitions of disease remission exists in clinical practice. I believe that to achieve complete remission it would be necessary to reach immunological remission.

Another controversial aspect in the management of RA is the concept of remission. According to recent recommendations, remission is a primary target of RA treatment [19]. However, the rate of remission of patients on biological drugs is only 20-30% in established disease [20].

The American College of Rheumatology (ACR) and EULAR have recently developed new remission criteria based on a Boolean approach or on an approach based on SDAI and CDAI criteria, which provide continuous numerical scales that reflect the activity of the disease and are closely related to the absence of residual inflammatory activity [19].

Furthermore, Baker et al. assert that remission as defined by ACR/EULAR is not the optimal particularly in clinical practice and in nonresearch settings and it does not always equate to absence of disease [21].

Consistent with this evidence, participants agreed that achieving full persistent disease remission is an unmet need in the treatment of RA.

Some reasons could be the absence of validated biomarkers that can measure remission [21] and the heterogeneity of the concept of remission as it may be interpreted from clinical, radiological, or immunological/molecular points of view.

Consistent with this heterogeneity, in 97% of cases participants identified both clinical and radiological remission as those preferentially used in clinical practice. Regarding the concept of immunological remission, almost 30% of the participants did not identify it as preferential, underlining how the definition of immunological remission is still unclear and is not supported by robust evidence. Although some evidence concerning the use of T-cell subset to objectify the phenotype of patients in remission has introduced the concept of immunological remission [20], objective criteria for defining remission that includes immunologic assessment of the disease state are absent [22] and the concept of molecular remission still remains elusive [10]. Furthermore, the 2016 Target Therapy Meeting identified the development of molecular definitions of disease remission as a primary unmet scientific need in RA [4].

## 5. Conclusion

Despite available therapies, many unmet medical needs are still present in the treatment of RA patients. By addressing them, we could improve patients' RA management. In particular, the high heterogeneity among both the patients and the disease as well as the poor achievement of clinical remission can lead to the observation that additional drugs will be needed to meet the needs of all patients and health care professionals.

## Figures and Tables

**Table 1:** Answers to the Delphi questionnaire (Items 1 and 2). The table shows the Delphi questionnaire and the answers relating to the individual items. The answers given by the Delphi participants are expressed in numerical terms. The percentages indicate the sum of the answers related to nonconsensus (1, 2) and consensus (3, 4, and 5).

**Optimal pain control**					
**1. Pain plays an increasingly important role in the patients' perception of the severity of the disease. Therefore, to obtain adequate pain control, I deem it is necessary to evaluate the following PROs (patient reported outcomes):**					

	**1**	**2**	**3**	**4**	**5**

1.1 Mood	**1**	**1**	**9**	**18**	**19**

	**4**%		**96**%		

1.2 Fatigue	**2**	**1**	**8**	**22**	**15**

	**6**%		**94**%		

1.3 Physical functioning	**2**	**1**	**6**	**16**	**23**

	**6**%		**94**%		

1.4 Sleep disturbances	**3**	**5**	**7**	**17**	**16**

	**17**%		**83**%		

**2. Current available therapies are not always able to improve pain. Selection of elements to consider for optimal pain control. Therefore, for optimal control of pain, I consider important:**					

	**1**	**2**	**3**	**4**	**5**

2.1 to achieve clinical remission	**1**	**0**	**0**	**17**	**30**

	**2**%		**98**%		

2.2 to use DMARDs for quick pain control	**1**	**1**	**4**	**24**	**18**

	**4**%		**96**%		

2.3 The use of appropriate anti-inflammatory analgesics, including the use of glucocorticosteroids at the lowest possible dose and for the shortest period as possible.	**1**	**0**	**4**	**20**	**23**

	**2**%		**98**%		

2.4 psychological / educational support provided by the doctor to the patient	**1**	**1**	**14**	**19**	**13**

*Abbreviations.* PROs: patient reported outcomes; DMARD: disease-modifying antirheumatic drug

**Table 2:** Answers to the Delphi questionnaire. (Items 3 and 4). The table shows the Delphi questionnaire and the answers relating to the individual items. The answers given by the Delphi participants are expressed in numerical terms. The percentages indicate the sum of the answers related to nonconsensus (1, 2) and consensus (3, 4, and 5).

**Significant fatigue improvement**					
**3. Fatigue continues to have a significant negative impact on over half of patients with RA, and it is a factor that impacts on patient's quality of life. I think that:**					

	**1**	**2**	**3**	**4**	**5**

3.1 Only in a certain percentage of patients fatigue is related to the progression of the disease	**1**	**8**	**15**	**19**	**5**

	**19**%		**81**%		

3.2 Fatigue is not always a valid indicator to evaluate the effectiveness of a therapy	**1**	**5**	**13**	**17**	**12**

	**13**%		**87**%		

3.3 The FACIT-fatigue (Functional Assessment of Chronic Illness – fatigue) in Italian is a valid index to monitor the fatigue	**1**	**2**	**24**	**17**	**4**

	**6**%		**94**%		

3.4 It is correct to include the extent of fatigue in clinical trial	**1**	**1**	**17**	**20**	**9**

	**4**%		**96**%		

3.5 Fatigue can correlate significantly with mood	**1**	**0**	**8**	**16**	**23**

	**2**%		**98**%		

**Satisfactory levels of physical functioning**					

**4. Physical functioning levels depend on disease activity, structural damage and fatigue. Current therapies do not often provide satisfactory levels of physical functioning Therefore to improve physical functioning I consider important:**					

	**1**	**2**	**3**	**4**	**5**

4.1 Intervening at an early stage with second-level drugs (bDMARDS o tsDMARDs)	**2**	**0**	**6**	**20**	**20**

	**4**%		**96**%		

4.2 Implementing a T2T strategy with closer monitoring	**1**	**0**	**4**	**19**	**24**

	**2**%		**98**%		

4.3 Improving adherence to therapy in order to have optimal control of the disease	**1**	**0**	**4**	**19**	**24**

	**2**%		**98**%		

4.4 Starting a joint education program immediately after the onset of the disease	**1**	**1**	**17**	**16**	**13**

	**4**%		**96**%		

4.5 Always administering a low dose of cortisone in association with DMARDs	**4**	**13**	**23**	**6**	**2**

	**35**%		**65**%		

4.6 Administering a low dose of cortisone in association with DMARD for a limited period	**1**	**4**	**12**	**14**	**17**

	**10**%		**90**%		

*Abbreviations.* RA: rheumatoid arthritis; FACIT-fatigue: Functional Assessment of Chronic Illness–fatigue; bDMARD: biologic disease-modifying antirheumatic drug; tsDMARD: targeted synthetic disease modifying anti-rheumatic drug; T2T: treat to target.

**Table 3:** Answers to the Delphi questionnaire. (Items 5 and 6). The table shows the Delphi questionnaire and the answers relating to the individual items. The answers given by the Delphi participants are expressed in numerical terms. The percentages indicate the sum of the answers related to nonconsensus (1, 2) and consensus (3, 4, and 5).

**Maintain workability **					
**5. One third of subjects with RA stop working at an early age. 30-40**%** of the patients show disability after 5 years of diagnosis. The desire to return to a normal working life is one of the main goals from the patient's perspective. I believe that to achieve this goal it is important:**					

	**1**	**2**	**3**	**4**	**5**

5.1 Diagnosis and early treatment to avoid disability	**1**	**0**	**1**	**8**	**38**

	**2**%		**98**%		

5.2 To Improve adherence to therapy	**1**	**1**	**4**	**18**	**24**

	**4**%		**96**%		

5.3 To include the preservation of productivity as an integral part of the therapeutic goals of treatment	**0**	**1**	**8**	**18**	**21**

	**2**%		**98**%		

5.4 To Evaluate the patient's work productivity / inability to work at least every six months in normal clinical practice	**0**	**3**	**16**	**19**	**10**

	**6**%		**94**%		

**Social Functioning **					

**6. Although the “desire for normality" is very important for patients with RA, the aspect of social functioning has never been the main focus of RA publications. Therefore I think that in order to take into account the patients' perspective, it is appropriate**					

	**1**	**2**	**3**	**4**	**5**

6.1 To include the return to a normal social life among the treatment goals	**0**	**1**	**7**	**24**	**16**

	**2**%		**98**%		

6.2 Use the SF-36 for global evaluation of the QoL	**1**	**3**	**23**	**16**	**5**

	**8**%		**92**%		

6.3 Include the evaluation of social functioning in the objectives of clinical trials	**0**	**2**	**18**	**20**	**8**

	**4**%		**96**%		

6.4 The sharing between doctor and patient of the possible impact of therapy on social life	**0**	**1**	**6**	**22**	**19**

	**2**%		**98**%		

*Abbreviations.* RA: rheumatoid arthritis; SF-36: short form 36; QoL: quality of life.

**Table 4:** Answers to the Delphi questionnaire. (Items 7 and 8). The table shows the Delphi questionnaire and the answers relating to the individual items. The answers given by the Delphi participants are expressed in numerical terms. The percentages indicate the sum of the answers related to nonconsensus (1, 2) and consensus (3, 4, and 5).

**treatment adherence **					
**7. Adherence to DMARD is often suboptimal in RA patients. Poor adherence can reduce the effectiveness of any treatment. Therefore I believe that the factors that can influence therapeutic adherence are:**					

	**1**	**2**	**3**	**4**	**5**

7.1 A shared decision between doctor and patient about drug treatment	**1**	**1**	**1**	**14**	**31**

	**4**%		**96**%		

7.2 The mode of administration of the drug	**0**	**3**	**14**	**19**	**12**

	**6**%		**94**%		

7.3 The frequency of administration of the drug	**1**	**3**	**15**	**16**	**13**

	**8**%		**92**%		

7.4 Rapid improvement of symptoms	**1**	**2**	**5**	**13**	**27**

	**6**%		**94**%		

7.5 Side effects	**1**	**0**	**4**	**15**	**28**

	**2**%		**98**%		

7.6 Patient involvement in specific programs	**1**	**4**	**18**	**11**	**14**

	**10**%		**90**%		

**Prognostic factors for treatment response**					

**8. There are no clear prognostic factors for response to therapies. in the choice of treatment I rely on:**					

	**1**	**2**	**3**	**4**	**5**

8.1 Patient's Phenotypic characteristics: age, sex, concomitant therapies, body mass index and life habits	**0**	**0**	**8**	**22**	**18**

	**0**%		**100**%		

8.2 The presence of any comorbidity	**1**	**0**	**2**	**15**	**30**

	**2**%		**98**%		

8.3 The prevalent biological pathway to choose the most suitable MoA drug by searching for specific biomarkers (SNPs, micro-RNA, polymorphisms, etc.)	**5**	**14**	**12**	**11**	**6**

	**40**%		**60**%		

8.4 The possible presence of anti-drug antibodies if the patient is a secondary failure	**6**	**17**	**9**	**10**	**6**

	**48**%		**52**%		

8.5 The MOA of biological drugs administered before	**0**	**5**	**9**	**18**	**16**

	**10**%		**90**%		

*Abbreviations.* DMARD: disease-modifying antirheumatic drug; RA: rheumatoid arthritis; MoA: mechanism of action; SNPs: single nucleotide polymorphism; RNA: ribonucleic acid.

**Table 5:** Answers to the Delphi questionnaire. (Item 9, 10 and 11). The table shows the Delphi questionnaire and the answers relating to the individual items. The answers given by the Delphi participants are expressed in numerical terms. The percentages indicate the sum of the answers related to non-consensus (1, 2) and consensus (3, 4 and 5).

**Best condition for an effective mono-therapy**					
**9. In “real life" a high percentage (30-50**%**) of patients that are undergoing combination therapy with bDMARD and methotrexate, only take bDMARD, which leads to an expected reduction in therapeutic efficacy. Therefore to avoid this you would need to:**					

	**1**	**2**	**3**	**4**	**5**

9.1 Preferentially use bDMARDs that are effective in monotherapy	**1**	**11**	**14**	**14**	**8**

	**25**%		**75**%		

9.2 Use tsDMARDs preferentially	**1**	**11**	**17**	**14**	**5**

	**25**%		**75**%		

9.3 Choose drugs with lower risk of onset of immunogenicity	**1**	**9**	**13**	**19**	**6**

	**21**%		**79**%		

9.4 Implement follow-up programs even at home	**2**	**6**	**16**	**20**	**4**

	**17**%		**83**%		

**Disease remission**					

**10. The “treat to target" approach has been proposed since 2010 for RA patients, with remission as the best treatment outcome possible. However, high heterogeneity in the definitions of disease remission exists in clinical practice. I believe that to achieve complete remission it would be necessary to reach:**					

	**1**	**2**	**3**	**4**	**5**

10.1 Clinical remission	**1**	**0**	**5**	**9**	**33**

	**2**%		**98**%		

10.2 Radiological remission (imaging)	**0**	**2**	**10**	**10**	**26**

	**4**%		**96**%		

10.3 Immunological remission	**3**	**9**	**15**	**11**	**10**

	**25**%		**75**%		

**11. In clinical practice, the state of the disease evaluation is subjected to various criteria. In assessing clinical remission I rely on:**					

	**1**	**2**	**3**	**4**	**5**

11. a.SDAI (simplified disease activity index) or CDAI (clinical disease activity index), Boolean remission	**1**	**2**	**14**	**15**	**16**

	**6**%		**94**%		

11.2 DAS28-ESR or DAS28-CRP	**1**	**3**	**11**	**16**	**17**

	**8**%		**92**%		

11.3 Ultrasonographic investigations	**3**	**3**	**17**	**11**	**14**

	**13**%		**87**%		

11.4 Composite indexes, which also take into account the patient's point of view (PROs)	**3**	**5**	**19**	**14**	**7**

	**17**%		**83**%		

*Abbreviations.* bDMARD: biologic disease-modifying antirheumatic drug; tsDMARD: targeted synthetic disease modifying antirheumatic drug; RA: rheumatoid arthritis; SDAI, Simplified Disease Activity Index; CDAI: Clinical Disease Activity Index; DAS-28-ESR: Disease Activity Score 28 erythrocyte sedimentation rate; DAS28-CRP: Disease Activity Score 28 C-reactive protein. PROs: patient reported outcomes.

## Data Availability

The authors hereby state that their paper refers to a Delphi exercise, so no original data are reported. The full story of the Delphi exercise can be found as a data file on the central server at “Tbwa Italia Spa, Via Privata Leto Pomponio, 3, 20146 Milano, Italy,” that provided technical assistance during the process.
